# National incidence, prevalence and disability-adjusted life years (DALYs) of common micronutrient deficiencies in Ethiopia from 1990 to 2017: estimates from the global burden of diseases study

**DOI:** 10.1080/16549716.2020.1776507

**Published:** 2020-07-02

**Authors:** Hamid Yimam Hassen, Jemal Haider Ali, Seifu Hagos Gebreyesus, Bilal Shikur Endris, Awoke Misganaw Temesgen

**Affiliations:** a Department of Public Health, Mizan Tepi University, Mizan Teferi, Ethiopia; b School of Public Health, Addis Ababa University, Addis Ababa, Ethiopia; c National Data Management Center, Ethiopian Public Health Institute, Addis Ababa, Ethiopia; d Institute for Health Metrics and Evaluation, University of Washington, Washington, DC, USA

**Keywords:** Incidence, prevalence, DALY, micronutrient deficiency, iodine, iron, vitamin A, Ethiopia

## Abstract

**Background:**

Understanding the national burden and trend of micronutrient deficiencies helps to guide effective intervention strategies under various circumstances. There is, however, a lack of evidence on trends, age- and sex-specific variations in Ethiopia.

**Objective:**

This study aimed to provide evidence on the trends of common micronutrient deficiencies including, dietary iron, iodine, vitamin A and other nutritional deficiencies in Ethiopia, from 1990 to 2017, using findings from the Global Burden of Disease study.

**Method:**

We used estimates from the GBD 2017 study to report the incidence, prevalence and disability-adjusted life years of micronutrient deficiencies in Ethiopia from 1990 to 2017. All estimates, both crude counts, as well as all-age and age-standardized rates per 100,000 population, are accompanied by 95% uncertainty intervals (UIs). We summarized the age- and sex-specific patterns and we compared the burden with the sub-Saharan Africa and global estimate.

**Results:**

From 1990 to 2017, the age-standardized prevalence rate of dietary iron, vitamin A and iodine deficiency decreased by 20.1%, 16.7%, and 91.6%, respectively. However, MNDs still account for a large number of DALYs in the country. In 2017, the all-age total DALYs due to dietary iron deficiency were estimated to be 448.4 thousand [95% UI: 298.9–640.7], accounting for 1.18% of the total DALYs. Similarly, the all-age total DALYs due to vitamin A deficiency were 397.8 thousand [256.1–589.2]. The total DALYs due to iodine deficiency were estimated to be 89.6 thousand [48.3–155.4].

**Conclusions:**

Micronutrient deficiencies and associated morbidity and mortality are still high in Ethiopia compared with the sub-Saharan and global estimate. Adolescent and early adult females and children aged under-five are disproportionately affected segments of the population. Therefore, in collaboration with other sectors, the National Nutrition Program needs to place greater emphasis upon improving accessibility and utilization of nutrient-rich foods and supplementation, particularly for vulnerable groups of the population.

## Background

Micronutrient deficiencies (MNDs) are an important contributor to the global burden of disease through increasing rates of illness and death from infectious diseases, and of disability such as mental impairment and vision loss. Globally, more than two billion people suffer from at least one form of micronutrient deficiency [1]. The burden prevails worldwide with adolescent girls and children under the age of five at highest risk. Common MNDs including iron, vitamin A, iodine, folate, and zinc deficiencies contribute to poor growth, intellectual disabilities in children, and perinatal complications leading to increased risk of morbidity and mortality [2,3].

Iron deficiency is the most common MND worldwide, affecting more than 30% of the world’s population [4], leading to microcytic anemia, decreased capacity to work, and impaired immune and endocrine functions [5–7]. The 2011 WHO report indicates anemia affects around 800 million children and women globally [8]. Similarly, iodine deficiency is also widespread, with approximately two billion people estimated to have inadequate iodine status globally [9], causing endemic goiter, mental retardation, or reduced cognitive function [10–12]. Vitamin A deficiency (VAD) is also associated with an increased rates and severity of infections and is the primary cause of childhood morbidity and mortality [13,14]. The WHO estimates, 250–500 million children are blind as a result of VAD, and half of these children die within a year of vision loss [15].

The burden of MNDs varies across regions and countries, with higher prevalence in sub-Saharan Africa (SSA) and South Asia, and the lowest in North America [16]. The higher prevalence in SSA could be due to the interaction of inadequate dietary diversity, poor sanitation and infectious diseases. Vitamin and mineral deficiencies affect more than one-third of SSA’s population, overwhelming both national healthcare and economic systems [17]. More than 20% of children under the age of five suffer from VAD in 37 countries of the African region including Ethiopia [18]. Similarly, the prevalence of anemia is more than 40% in the majority of African countries, and 13 countries currently have an iodine deficiency rate of over 50% among school-aged children [19].

In Ethiopia, MNDs are common nutritional problems affecting all segments of the population, particularly the under-fives and pregnant women. The Ethiopian Demographic and Health Survey showed that the prevalence of anemia among under-fives in 2005, 2011 and 2016 were 54%, 44% and 57%, respectively [20–22], indicating no decrease despite the implementation of various intervention strategies. The 2016 National Micronutrient Survey indicated that the prevalence of iodine deficiency is high among school-age children and women of childbearing age, in which 47.5% and 51.%, respectively had urinary iodine levels less than 100 μg/L [23]. Similarly, a review article conducted in 2014 showed that severe iodine deficiency among Ethiopian women leads to 50,000 stillbirths annually and the country’s goiter rate ranges from 14% to 59% [24].

Cognizant of the problem, several strategies have been implemented to reverse the increasing trend of MND such as universal salt iodization, Vitamin A supplementation for children, iron/folate supplementation for pregnant women, and Infant and Young Child Feeding (IYCF) program. However, MNDs remain a major public health problem in Ethiopia. Determining the burden and trends of MNDs is critical to understand the status of intervention strategies in various contexts and at various time periods. Therefore, this study aimed to summarize the trend and current burden of micronutrient deficiencies in Ethiopia, including sex- and age-specific variations, and to compare with the SSA and global estimate using the 2017 Global Burden of Diseases, Injuries, and Risk Factors Study (GBD 2017).

## Methods

### GBD 2017

The GBD 2017 estimated the incidence, prevalence, YLLs, YLDs, and DALYs for 354 diseases and 195 countries from 1990 to 2017. The GBD 2017 methods including case definitions, data sources, and estimation process are described in extensive detail elsewhere [25]. The methods used specific to micronutrient deficiencies are briefly summarized here.

### Case definitions

In GBD 2017 study, dietary iron deficiency was estimated from all forms; mild, moderate and severe iron deficiency anemia. VAD assessment involves the quantification of total VAD (serum retinol < 0.7 µmol/L) as well as blindness and vision loss due to VAD, which are associated with corneal ulcerations and corneal scars. Nonfatal burden of iodine deficiency was estimated using visible goiter (grade 2) only and its associated sequelae, such as thyroid dysfunction, heart failure, and intellectual disability. It does not include sub‐clinical iodine deficiency or non‐visible goiter (grade 1). Other nutritional deficiencies encompass a wide variety of causes of morbidity, ranging from mineral and vitamin deficiencies to other nutritional anemia. Due to their relatively limited burden, diversity in underlying causes and risk factors, and data availability, GBD 2017 treats these causes as a single category.

### Data sources

Data from the WHO Vitamin and Mineral Nutrition Information System database, Ethiopian Demographic and Health Surveys 2005–2016, the Office of US Foreign Disaster Assistance/Center for Research on the Epidemiology of Disasters database, several surveys in different parts of Ethiopia, and systematic reviews of published studies were used. To estimate VAD, additional data sources, including Micronutrient Surveys (MNS) and Multiple Indicator Cluster Survey (MICS) were also considered.

### Incidence, prevalence and DALY estimation process

The 2017 GBD study modelled the prevalence of micronutrient deficiencies using DisMod‐MR 2.1 [26]. It is a Bayesian meta-regression tool which combines epidemiological data from multiple sources, adjusts inconsistent data and forecasts and updates data for regions and parameters with no or little data. To model iodine deficiency, grade 2 goiter was chosen over grade 1 due to the greater reliability and consistency of the clinical diagnosis of grade 2 goiter worldwide [27]. The modeling used a study‐level covariate to indicate national observations, where nationally representative studies were set as the reference category and household iodized salt consumption proportion as a country‐level covariate. The VAD estimates were made sequentially. *First*, vitamin A supplementation coverage was estimated. The case definition for the supplementation model was the proportion of children 6–59 months of age who received at least one dose of vitamin A in the previous 6 months. *Second*, age- and sex-specific prevalence of VAD (serum retinol < 0.7 µmol/L) was estimated. *Third*, for models of the prevalence of blindness and vision loss due to VAD, this was run as a single-parameter meta-regression on prevalence, so incidence estimates were not generated. Further details on modeling strategy is available elsewhere [25].

Disability-adjusted life-year (DALY) were calculated by summing years of life lost (YLLs) due to premature mortality and years of life lived with disability (YLDs), thereby incorporating both fatal and non-fatal burden. As there was no mortality data, all DALY estimates for dietary iron, vitamin A and iodine deficiency were from the YLDs.

All parameter estimates generated in the GBD 2017 were accompanied by 95% uncertainty intervals (UIs). The uncertainty ranges reported around YLDs incorporate both uncertainty in prevalence and in the disability weight. To do this, 1,000 samples of comorbidity-corrected YLDs and 1,000 samples of the disability weight to generate 1,000 samples of the YLD distribution were considered. The 95% uncertainty interval is reported as the 25th and 975th values of the distribution.

## Presentation and interpretation of results

We followed the GBD 2017 categorization of diseases and injuries to present and interpret the national incidence, prevalence, and DALY rates of common MNDs in Ethiopia from 1990 to 2017. We extracted the age- and sex-specific estimates from the GBD 2017 study, particularly for Ethiopia and compared it with the SSA and global estimates. We reported the burden of iodine, dietary iron, vitamin A and other nutritional deficiencies, including vitamin B12, folate, dietary zinc, calcium, vitamin D, thiamine, niacin, and vitamin C. We summarized the trends of age-standardized rates from 1990 to 2017 in terms of DALY, prevalence and incidence rate whenever available. We computed changes and reported positive and negative percentages to show an increasing and decreasing trend, respectively, from 1990 to 2017. We present the estimates in terms of the total number of DALYs, prevalent and incident cases, all-age and age-standardized rates using tables and figures. The results are reported in accordance with the Guidelines for Accurate and Transparent Health Estimates Reporting (annex 1).

## Results

### Burden of dietary iron, vitamin A, iodine and other nutritional deficiencies in Ethiopia in 2017

In 2017, the total DALYs due to dietary iron, vitamin A, iodine, and other nutritional deficiencies were estimated to be 448.4 thousand DALYs [95%UI: 298.9–640.7], 397.8 thousand [256.1–589.2], 89.6 thousand (48.3–155.4) and 19.0 thousand [13.3–24.6], respectively. These indicate 1.18% [95% CI: 0.83–1.60], 1.05% [0.71–1.50], 0.24% [0.13–0.39], and 0.05% [0.036–0.064] of all DALYs in the same year were attributable to dietary iron, vitamin A, iodine and other nutritional deficiencies, respectively. The prevalence of dietary iron, vitamin A and iodine deficiency was 14.4% [95% CI: 13.7–15.4], 28.0% [25.4–31.0], and 6.8% [6.1–7.6], respectively. The incidence rate of vitamin A and iodine deficiency were 23,295.3 per 100,000 [21,104.5–25,199.4] and 266.4 [237.7–299.1], respectively. The all-age rate, age-standardized rate and total DALYs, prevalence and incidence is summarized in Table 1.

**Table 1.** DALYs, prevalence and incidence of iodine, vitamin A, dietary iron and other nutritional deficiencies in Ethiopia, 1990 and 2017.

**DALYs**									
	**Age-standardized DALYs per 100,000**			**All-age DALYs per 100,000**			**All-age total DALYs (thousands)**		
Micronutrient deficiencies	1990	2017	% Change	1990	2017	% Change	1990	2017	% Change
Dietary iron deficiency	518.2 (346.2–744.8)	364.2 (245.3–522.0)	−29.7%	576.9 (383.6–829.4)	438.8 (290.5–622.7)	−23.9%	296.5 (197.2–426.3)	448.4 (298.9–640.7)	51.2%
Vitamin A deficiency	397.4 (256.9–585.5)	253.6 (162.5–376.6)	−36.2%	690.3 (446.4–1,016.4)	386.7 (248.9–572.6)	−44.0%	354.8 (229.5–522.5)	397.8 (256.1–589.2)	12.1%
Iodine deficiency	959.8 (474.8–1,727.5)	90.7 (48.7–157.4)	−90.6%	971.0 (480.9–1,749.4)	87.1 (47.0–151.0)	−91.0%	499.2 (247.2–899.3)	89.6 (48.3–155.4)	−82.0%
^a^Other nutritional deficiencies	182.4 (123.8–262.9)	22.7 (15.9–30.3)	−87.6%	184.0 (118.8–256.2)	18.4 (12.9–23.9)	−90%	94.6 (61.1–131.7)	19.0 (13.3–24.6)	−79.9%
**Prevalence**									
Micronutrient deficiencies	**Age-standardized prevalence per 100,000**			**All-age prevalence per 100,000**			**All-age total number of cases (thousands)**		
	1990	2017	% Change	1990	2017	% Change	1990	2017	% Change
Dietary iron deficiency	16,496.6 (15,142.3–18,017.9)	13,180.6 (12,581.6–13,901.3)	−20.1%	16,432.3 (15,086.2–18,090.8)	14,444.4 (13,714.3–15,383.7)	−12.1%	8,446.9 (7,755.0–9,299.5)	14,861.0 (14,109.8–15,827.4)	75.9%
Vitamin A deficiency	31,598.3 (28,704.1–34,349.3)	26,237.4 (23,740.5–28,732.1)	−16.7%	35,037.7 (31,666.9–38,240.6)	28,020.7 (25,351.0–30,861.5)	−20.0%	18,011.0 (16,278.2–19,657.4)	28,828.8 (26,082.1–31,751.5)	60.1%
Iodine deficiency	85,989.9 (77,476.6–90,634.1)	7,257.3 (6,497.2–8,118.3)	−91.6	85,962.9 (77,409.0–90,631.6)	6,788.1 (6,051.7–7,633.8)	−92.1%	44,188.9 (39,791.8–46,588.9)	6,983.9 (6,226.2–7,853.9)	−84.2%
**Incidence**									
Micronutrient deficiencies	**Age-standardized incidence per 100,000**			**All-age incidence per 100,000**			**All-age total number of incident cases (thousands)**		
	1990	2017	% Change	1990	2017	% Change	1990	2017	% Change
Vitamin A deficiency	21,392.6 (19,151.5–23,524.0)	23,376.2 (21,025.0–25,377.3)	9.3%	20,911.7 (18,885–22,862.6)	23,295.3 (21,104.5–25,199.4)	11.4%	10,749.6 (9,707.8–11,752.5)	23,967.2 (21,713.1–25,926.2)	123%
Iodine deficiency	1,984.3 (1,786.2–2,090.9)	169.5 (151.8–189.7)	−91.5%	4,675.2 (4,203.3–4,929.6)	266.4 (237.7–299.1)	−94.3%	2,403.2 (2,160.7–2,534.0)	274.1 (244.6–307.7)	−88.6%

^a^Thiamine, niacin, other B group vitamins, ascorbic acid, vitamin D, other vitamin, dietary calcium, dietary selenium, dietary zinc, and other nutrient element deficiencies.

Data in parentheses are 95% uncertainty intervals. DALY = disability-adjusted life-year.

### Trends of dietary iron, vitamin A, iodine and other nutritional deficiencies from 1990 to 2017

The change in DALYs, prevalence and incidence of common micronutrient deficiencies in Ethiopia from 1990 to 2017 is summarized in Table 1. From 1990 to 2017, the age-standardized DALY rate due to iron, vitamin A, iodine and other nutritional deficiencies decreased by 29.7%, 36.2%, 90.6%, and 87.6%, respectively. However, all-age total DALYs due to dietary iron and vitamin A deficiency increased by 51.2% and 12.1%, respectively. The proportion of total DALYs attributable to dietary iron deficiency increased from 0.5% [95% UI 0.34–0.7] to 1.18% [0.83–1.6]. Similarly, the proportion of DALYs due to VAD increased from 0.6% [95% UI: 0.4–0.86] to 1.05% [0.71–1.5]. Whereas, the proportion of DALYs attributable to iodine deficiency declined dramatically from 0.85% [95% UI: 0.43–1.48] to 0.24% [0.13–0.39]. Likewise, the proportion of total DALYs attributable to other nutritional deficiencies decreased from 0.16% [95% UI: 0.1–0.22] to 0.05% [0.036–0.064].

**Figure 1.**
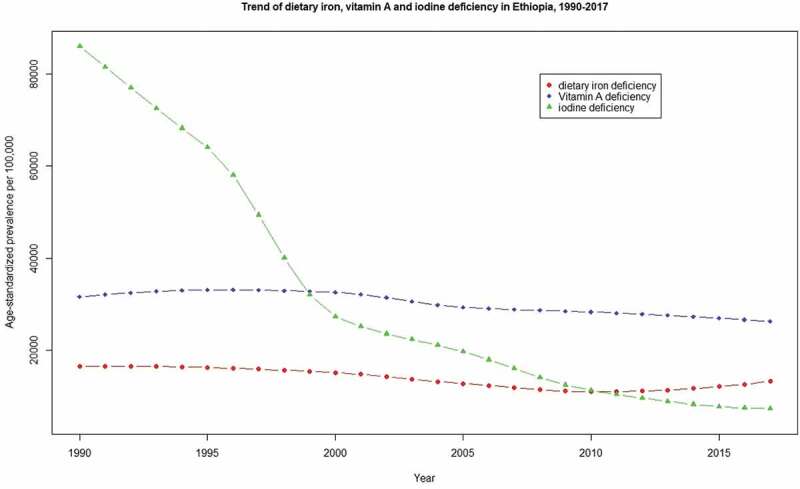
Trend of age-standardized dietary iron, vitamin A, and iodine deficiency prevalence in Ethiopia, from 1990 to 2017.

The age-standardized prevalence rate of dietary iron, vitamin A and iodine deficiency over this time period declined by 20.1%, 16.7%, and 83.6%, respectively. However, the age-standardized incidence rate of VAD has increased by 9.3%. The trend of age-standardized prevalence rate of dietary iron, vitamin A and iodine deficiency from 1990 to 2017 is summarized in Figure 1.

In 1990, iodine, vitamin A and dietary iron deficiency, respectively, were the 1st, 2nd, and 3rd causes of YLDs from all causes in Ethiopia. Whereas in 2017, they were ranked as the 3rd, 6th and 32nd-leading causes of YLDs, respectively. (Supplementary material)

### Sex-specific patterns

Detailed results of age and sex-specific patterns of DALYs, prevalence and incidence are available in the supplementary material. Overall, the burden of MNDs is higher among females than males. In 2017, the age-standardized DALY rate due to dietary iron deficiency among females and males was 422.2 per 100,00 [95%UI: 282.8–610.7] and 303.2 [199.7–440.4], respectively. Similarly, the age-standardized DALY rate of VAD was 295.7 per 100,00 [95%UI: 184.4–453.2] in females and 213.4 [136.9–318.0] in males. Further, the age-standardized DALY rate of iodine deficiency among females and males was 110.3 per 100,000 [58.0–193.5] and 71.2 [95%UI: 38.5–122.3] respectively. Moreover, the age-standardized DALY rate of other nutritional deficiencies was 26.8 per 100,000 [16.6–36.9] in females and 18.8 [13.0–42.1] in males.

### Age-specific patterns

**Figure 2.**
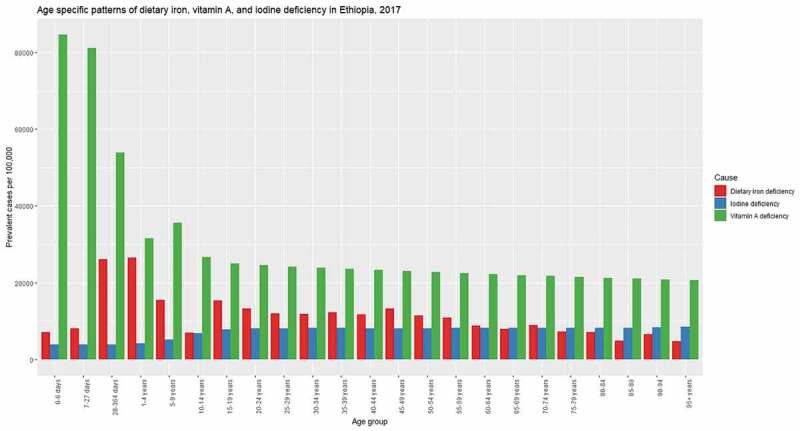
Age specific patterns for prevalence rate of dietary iron, vitamin A, and iodine deficiency in Ethiopia, 2017.

Overall, the burden of MNDs was higher among children and adolescents. In 2017, the highest prevalence rate of dietary iron deficiency was among children aged 1 to 4 years with 26,463.6 prevalent cases per 100,000 [95%UI: 25,297.8–27,577.6]. The highest prevalence rate of VAD was among early neonates with 84,547.1 per 100,000 [95%UI: 62,982.6–96,665.2], whereas, the highest incidence was among children aged 1 to 4 years with 24,296.4 new cases per 100,000 [95%UI: 21,603.6–26,817.3]. The prevalence rate of iodine deficiency is increasing with age. The highest number of prevalent cases were among adolescents aged 15 to 19, with 926.1 thousand prevalent cases [95%UI: 829.8–1,042.7]. However, the highest incidence was among children aged 5 to 9 years, with 304.1 new cases per 100,000 [95%UI: 238.9–373.5]. In 2017, 5.3 thousand [95%UI: 3.3–8.1] DALYs due to other nutritional deficiency were among children aged 1 to 4 years. Figure 2 summarized the prevalence rate of dietary iron, vitamin A and iodine deficiency across various age groups. Detailed results are presented in the supplementary material.

### Comparison with the SSA and global estimate

**Figure 3.**
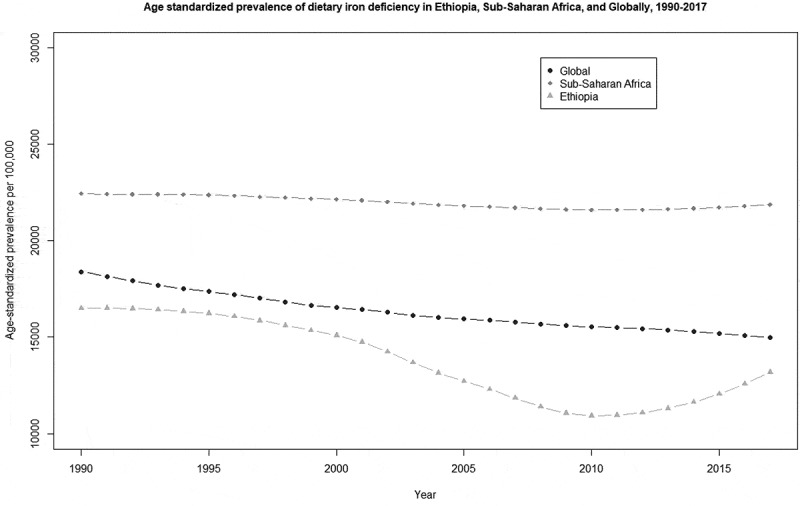
Comparison of age-standardized prevalence of dietary iron deficiency trend in Ethiopia, Sub-Saharan Africa, and globally, from 1990 to 2017.

Figure 3 shows the trend of dietary iron deficiency in Ethiopia, SSA and globally from 1990 to 2017. The figure shows a subtle decreasing trend in both SSA and globally. In Ethiopia, there was a decreasing trend from 1990 to 2010, but with a rise in 2017. In 2017, the prevalence rate in Ethiopia was lower than both the SSA and the global estimates.

**Figure 4.**
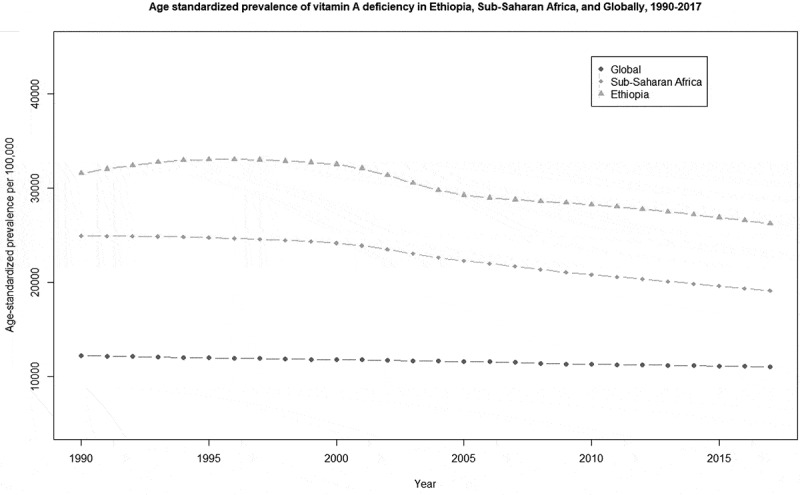
Comparison of age-standardized prevalence of vitamin A deficiency trend in Ethiopia, Sub-Saharan Africa, and globally, from 1990 to 2017.

Figure 4 shows the trend of VAD in Ethiopia, SSA and the globe from 1990 to 2017. The figure shows a downward trend in Ethiopia, SSA and globally. The prevalence of VAD in Ethiopia is consistently higher than the estimates from the SSA and the globe. In 2017, Ethiopia had the second highest prevalence of iodine deficiency of any country in the world.

**Figure 5.**
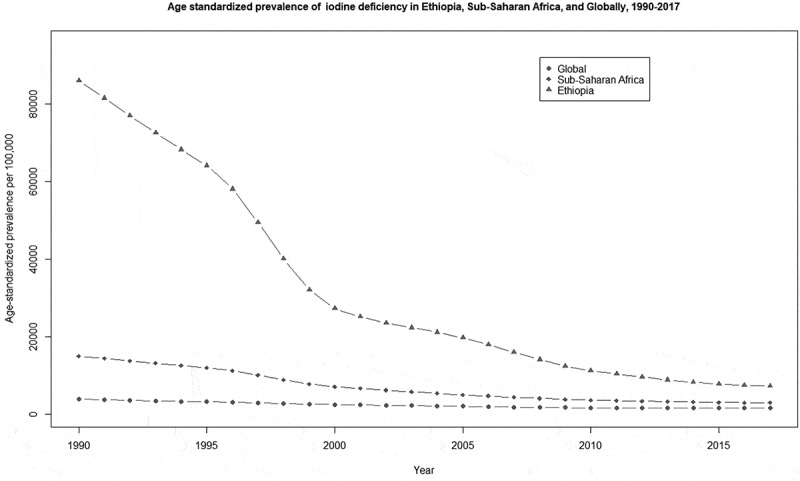
Comparison of age-standardized prevalence of iodine deficiency trend in Ethiopia, Sub-Saharan Africa, and globally, from 1990 to 2017.

Figure 5 shows the trend of iodine deficiency in Ethiopia, SSA and the globe from 1990 to 2017. We documented a decreasing trend in Ethiopia, SSA, and globally. In 2017, the prevalence rate in Ethiopia was much higher than the SSA and global estimate.

## Discussion

This study comprehensively describes the burden of micronutrient deficiencies including dietary iron, vitamin A, iodine and other nutritional deficiencies in Ethiopia, which allows a direct comparison over time, as well as between sex and age groups. Despite a decreasing trend in prevalence, MNDs still cause a remarkable burden in the country making them among the top causes of YLDs.

The overall prevalence of iodine deficiency in 2017 was 6.8%, indicating it is still an important public health problem. Several pilot studies in the country reported 16% to 62.1% total goiter rate (TGR) [28–32]. These studies, however, mainly focused on high-risk areas of the country and high-risk groups such as school children and women, and most of them used all stages of goiter, which resulted in higher prevalence rates. The prevalence of iodine deficiency greatly varies across regions due to geographical variation, diversities in food items and feeding habits, access to iodized salt, and access to iodine supplementation. Moreover, the variation in measurement of iodine deficiency, using either urinary iodine level or clinical examination of symptomatic goiter, could explain such a discrepancy.

A dramatic decline was observed in the age-standardized incidence and prevalence of iodine deficiency from 1990 to 2017. This could be due to the salt iodization program, which was started three decades previously. Ethiopia started iodization of salt in 1988 and banned the production and sale of non-iodized salt in 1996, until its interruption in 2000 due to the Ethio-Eritrea war [33,34]. From 2011, the universal salt iodization (USI) program became mandatory again [35]. According to the 2015 survey, conducted by the Ethiopian Public Health Institute (EPHI), 84.6% of salts in the country contain iodine [23]. However, the national USI program needs to improve the coverage of iodized salt at household level and in the remote areas of the country, where coverage is still very low. Only 26% of the total households were getting the required amount (more than 15 ppm) iodine in salt [23]. In 2017, the prevalence rate of iodine deficiency in Ethiopia was higher than the SSA and the global estimate, making the country with the highest iodine deficiency worldwide, second only to South Sudan. More than 17.5% of intellectual disability in the country are attributable to iodine deficiency [25].

This study showed that females, particularly adolescents and young adults, are disproportionately affected by higher incidence and prevalence of iodine deficiency. Several studies in many parts of the country also found such sex variation [28,30,32]. The difference could be due to androgen hormone in males which is stimulatory while estrogen hormone in females has an inhibitory effect on thyroid growth [36]. Moreover, the age at maximum thyroid growth (11–14 years), coinciding with menarche in girls, might contribute to a higher incidence of iodine deficiency during mid- to late puberty [37]. The highest number of iodine deficiency cases was observed in the age group of 10 to 19. This could be due to the increase in demand for iodine during puberty, which cannot be sufficient with the limited amount of iodine available in food and salt [38]. Further, the high prevalence in females has a devastating effect on maternity, and can lead to abortion, stillbirth, congenital anomalies, neurological cretinism, and increased perinatal and infant mortality.

The age-standardized prevalence of dietary iron deficiency in Ethiopia declined from 1990 to 2010 and rose in 2017. The decrease in consumption of animal products, due to an increase in cost since the Ethiopian Millennium (2008 GC), might be the possible reason. A review by Birhanu AF indicated, the total meat production in Ethiopia increased from 2004 to 2014 but decreased until 2017 [39]. Furthermore, the meat export rate increased from 2000 to 2019 [39,40], which could have an impact on the domestic meat cost and consumption. In 2017, one-fifth of the total population had dietary iron deficiency. Despite several programs addressing such issues in the last two decades, dietary iron deficiency is still a significant public health issue that needs integrated interventions. When iron intake no longer meets the need of normal iron turnover and losses, iron deficiency anemia will occur [41], a phenomenon which accounts for 50% of anemia globally [42].

The present study showed sex variation in the burden of dietary iron deficiency, the prevalence in females is higher than males. Bone marrow radiolabeled iron (Fe) studies showed iron absorption is dependent on body iron stores and is not gender-specific [43]. This indicates the sex difference in Ethiopia might not be explained by physiological differences but rather could be due to gender differences in dietary intake of iron rich foods. Besides, menstruation could also lead to negative iron balance in apparently healthy females [44]. The public health impact of iron deficiency is more pronounced in females as it results in an intergenerational deficiency in the future offspring unless timely measures are taken.

Overall, the prevalence of dietary iron deficiency is higher among children under the age of five. Despite an increase in breastfeeding rates [45], improvement in maternal and childcare services, and supplementation of iron [46], the prevalence of dietary iron deficiency anemia remains high among children. This could be due to the lower coverage of optimal dose of iron supplementation in the country. A study conducted by Gebremedhin S. and his colleagues found, only 3.5% of pregnant women took the required dose of iron [47]. Moreover, prevalence in infancy and childhood could be attributed to higher rates of low birth weight, preterm birth and maternal iron depletion during pregnancy [22]. The age distribution in dietary iron deficiency varies among males and females. In males, the highest prevalence is observed among children under the age of five, while, in females, late adolescent and early adults are the most affected age groups. The higher prevalence rates amongst late adolescent and early adult females could be due to menstrual bleeding and periods of rapid growth [48–50].

In this study, the prevalence of VAD in 2017 is estimated to be 28%, making Ethiopia the country with the second highest prevalence in the world. A study conducted by Semba RD. showed only 46.8% of children aged 12–59 months received vitamin A capsule [51]. Several studies showed a lack of vitamin A weakens the immune system and is associated with high rates of morbidity and mortality among under-fives [52–55]. VAD is also reported as the leading cause of preventable child blindness.

The prevalence of VAD is almost similar among males and females. There are, however, large variations across age groups, in which early and late neonates are the most affected group. In general, higher prevalence is observed in children, which increases susceptibility to infection, leading to increased rates of morbidity and premature death. Ethiopia launched a vitamin A supplementation program for under-fives in 2008 but the prevalence of VAD remains constantly higher than the SSA and the global estimate, which emphasizes the need for a better strategy to halt the incidence and associated morbidity and mortality.

## Limitations

Despite using estimates from GBD study in which robust methods of data collection and analysis were utilized, the estimates of this study should be interpreted within the context of the following limitations. Detailed methodological considerations of the overall estimation process for non-fatal outcomes have been discussed elsewhere [25]. Limitations specific to these estimates are discussed here. First, lack of availability of good quality data particularly to estimate incidence and prevalence of other nutritional deficiencies including zinc, calcium, folate, vitamin B12, vitamin C, etc. Second, the separate estimation of non-fatal models in the modeling process for 1990, 1995, 2000, 2006, 2010, and 2017 implies an uncertainty of estimates over time is independent. Third, accessing of accurate information on population coverage for any given data source is still a challenge. Fourth, due to the absence of mortality data, the DALYs were from YLDs only, which might lead to underestimation. Lastly, iodine deficiency was estimated using only grade 2 goiter, which might underestimate the prevalence. Including all forms of iodine deficiency is a goal of future iterations of the GBD study. Despite these limitations the present study provides a comprehensive estimate using the available evidences in the country.

### Implications of the estimates for policy and practice

These estimates are important for observing and evaluating the performance of previous nutritional intervention programs that have been implemented in Ethiopia and to support the current national nutrition strategic plan. Furthermore, these estimates could be utilized to scale up the existing strategies and programs in the field of nutrition or to design a better approaches for intervention. The disproportionate high burden of micronutrient deficiency among females and children aged under 5 years, needs further causal assessment and targeted intervention. The higher prevalence among women of child-bearing age and under-fives could have a substantial impact on health and behavioral outcomes over the lifespan and future generations in the country. Therefore, programs that are working on nutrition improvement should give more priority to these age groups.

### Progresses in combating the problems

Significant progress is observed in reducing iodine deficiency in all ages from 1990 to 2017 globally but Ethiopia remains second highest in iodine and vitamin A deficiency worldwide. There might be regional variation within the country, though our estimates did not capture the sub-national discrepancies. The trend of dietary iron deficiency is mixed, there was little improvement until 2010, thereafter showing an increment. This calls for a better intervention approach to combat dietary iron deficiency and associated complications leading to death. There is a decreasing trend in the burden of vitamin A deficiency, though it is minimal. Likewise, whilst a decreasing trend is observed in the burden of other nutritional deficiencies, the estimate is less precise due to a lack of good quality data.

## Conclusions

Despite the efforts made for the last few decades, the prevalence of dietary iron deficiency and associated morbidity and mortality are still high in Ethiopia. Females at adolescence and early adulthood and children under the age of five are disproportionately affected groups, which calls for targeted intervention. Similarly, no significant improvement has been observed in the prevalence of Vitamin A deficiency in Ethiopia in the last two decades, and the country is still has the second highest prevalence in the world. Therefore, apart from supplementation programs, agricultural and nutritional efforts should be made to improve access and production of micronutrient-dense foods. The availability and accessibility to animal-source foods that contain highly bioavailable micronutrients, particularly iron, vitamin A, zinc, and vitamin B12, need to be improved through policy measures and education. The National Nutrition Program needs to make more effort to improve accessibility and utilization of nutrient-rich foods in collaboration with other sectors including the agricultural sector. Further studies are needed to assess the causal association of various determinant factors of micronutrient deficiencies. Moreover, implementation research, to evaluate the effectiveness of several intervention approaches, pilot and scale up the best practice, is highly recommended.

## Supplementary Material

Supplemental Material

## Paper context

Understanding the burden of micronutrient deficiencies is essential to guide effective intervention strategies. However, there is a lack of comprehensive estimates in Ethiopia to direct policy. This article provides the trends of micronutrient deficiencies in Ethiopia, age- and sex-specific patterns, and comparison with the Sub-Saharan Africa and global estimates. The evidence helps policymakers to reconsider the national nutrition plan aiming at targeted intervention and intersectoral collaboration to improve nutritional values of staple foods in the country.
