# Supplementary material for: National incidence, prevalence and disability-adjusted life years (DALYs) of common micronutrient deficiencies in Ethiopia from 1990 to 2017: estimates from the global burden of diseases study
Source: Glob Health Action. 2020 Jul 2;13(1):1776507. doi: 10.1080/16549716.2020.1776507 (PMC7480576; doi:10.1080/16549716.2020.1776507)

Supplementary content:

National incidence, prevalence and disability adjusted life years (DALYs) of common micronutrient deficiencies in Ethiopia from 1990 to 2017: estimates from the Global Burden of Disease study

Hamid Yimam Hassen*[1], Jemal Haider Ali[2], Seifu Hagos Gebreyesus[2], Bilal Shikur Endris[2], Awoke Misganaw Temesgen[3,4]

This supplementary content provides age and sex specific patterns of incidence, prevalence, and DALYs of dietary iron, vitamin A, iodine, and other nutritional deficiencies in Ethiopia, 2017. Some of the results from this figure are presented in the main manuscript.

Table 1: Age and sex specific Disability Adjusted Life Years (DALYS) due to dietary iron, vitamin A, iodine and other nutritional deficiencies in Ethiopia, 2017

Dietary iron deficiency						
	**DALY rate per 100,000 (95%UI)**			**Total number of DALYs (95%UI)**		
**Age**	**Male**	**Female**	**Total**	**Male**	**Female**	**Total**
0-6 days	303.2 (194.9–450.7)	269.5 (179.7–386.4)	286.8 (192.1–417.2)	109.7 (70.5–163.1)	92.1 (61.4–132.0)	201.8 (135.1–293.5)
7-27 days	374.5 (244.4–561.8)	265.3 (176.8–381.0)	321.3 (214.8–470.8)	399.9 (260.9–599.8)	269.0 (179.2–386.3)	668.8 (447.2–980.0)
28-364 days	1,295.8 (849.1–1,913.6)	816.9 (547.1–1,178.4)	1,062.2 (706.9–1,543.8)	21,603.2 (14155.4–31903.1)	12,973.4 (8,688.6–18,715.6)	34,576.6 (23,010.7–50,255.9)
1-4 years	982.0 (653.8–1,418.8)	850.4 (541.7–1,241.4)	917.7 (596.1–1,333.8)	65,763.8 (43,782.2–95,011.8)	54,388.1 (34,647.5–79,392.4)	120,151.9 (78,044.2–174,629.0)
5-9 years	746.6 (448.8–1,143.7)	486.3 (289.0–801.0)	619.6 (390.6–928.6)	56,666.9 (34,062.7–86,801.2)	35,158.1 (20,892.2–57,910.1)	91,825.0 (57,885.9–137,619.0)
10-14 years	288.5 (111.9–654.9)	212.3 (92.3–516.8)	251.4 (121.3–474.5)	20,319.8 (7,880.2–46,129.6)	14,206.3 (6,175.5–34,574.3)	34,526.1 (16,657.4–65,163.9)
15-19 years	194.7 (113.3–313.7)	442.3 (279.1–673.6)	316.4 (201.4–471.6)	11,814.9 (6,875.7–19,032.5)	25,943.9 (16,375.0–39,511.4)	37,758.8 (24,033.0–56,281.4)
20-24 years	115.5 (68.4–188.1)	574.7 (371.0– 830.0)	345.6 (223.0–500.6)	5,800.4 (3,435.6–9,446.4)	28,987.0 (18,710.9–41,864.2)	34,787.4 (22,449.2–50,388.2)
25-29 years	117.9 (66.7–205.7)	478.6 (314.0– 707.0)	303.4 (198.1–446.6)	4,548.5 (2,574.4–7,938.5)	19,547.2 (12,822.9–28,873.0)	24,095.7 (15,737.8–35,477.9)
30-34 years	92.3 (51.5–154.5)	535.6 (351.2–783.6)	323.4 (212.0–471.3)	2,847.1 (1,587.9–4,765.8)	18,000.3 (11,802.9–26,334.2)	20,847.4 (13,664.7–30378.8)
35-39 years	142.1 (79.0–227.7)	399.9 (259.5–594.4)	275.5 (177.9–409.9)	3,770.3 (2,097.4–6,041.7)	11,383.3 (7,386.8–16,919.1)	15,153.6 (9,786.9–22,546.2)
40-44 years	151.1 (86.4–248.0)	341.4 (216.8–509.3)	245.0 (157.7–366.1)	3,273.2 (1,872.9–5,374.4)	7,347.8 (4,667.0–10,961.9)	10,621.0 (6,811.6–15,811.1)
45-49 years	184.9 (101.8–303.5)	342.8 (220.0–531.7)	261.4 (162.8–397.5)	2,994.6 (1,649.1–4,914.5)	5,211.8 (3,344.7–8,082.2)	8,206.4 (5,110.0–12,478.8)
50-54 years	199.2 (117.0–342.7)	211.7 (74.0–465.8)	205.3 (108.7–364.6)	2,399.8 (1,409.3–4,129.7)	2,436.2 (850.9–5,360.0)	4,836.0 (2,560.0–8,588.1)
55-59 years	156.7 (83.3–284.7)	237.4 (96.9–522.8)	196.6 (99.2–365.9)	1,423.4 (7,56.8–2,586.6)	2,112.2 (861.6–4,650.5)	3,535.6 (1,784.2–6,580.3)
60-64 years	132.9 (46.5–314.0)	204.3 (78.0–502.8)	168.3 (76.6–356.0)	957.9 (335.2–2,263.0)	1,443.7 (551.00– 3,552.61)	2,401.6 (1,093.3–5,081.8)

65-69 years	70.8 (25.1–176.2)	282.4 (116.6–591.4)	171.9 (76.8–338.9)	394.5 (139.6–981.6)	1,439.8 (594.7–3,015.2)	1,834.3 (820.0–3,615.6)
70-74 years	122.1 (39.6–295.3)	213.7 (85.1–452.6)	165.5 (76.3–322.6)	502.1 (162.9–1,213.9)	792.4 (315.4–1,678.3)	1,294.5 (596.7–2,522.6)
75-79 years	131.6 (47.0–302.2)	91.9 (23.7–277.6)	112.5 (44.2–240.3)	352.1 (125.7–808.5)	228.6 (58.8–690.2)	580.7 (228.1–1,240.4)
80+	83.9 (39.3–167.0)	162.7 (62.8–404.6)	121.2 (61.2–234.8)	181.5 (85.0–361.3)	316.7 (122.3–787.8)	498.2 (251.5–965.2)
Total	397.2 (262.0–576.2)	475.2 (317.6–689.7)	435.8 (290.5–622.7)	206,123.7 (135,996.9–299,019.0)	242,277.7 (161,940.5–351,656.8)	448,401.4 (298,881.5–640,699.9)

Vitamin A Deficiency

	**DALY rate per 100,000 (95%UI)**			**Total number of DALYs (95%UI)**		
**Age**	**Male**	**Female**	**Total**	**Male**	**Female**	**Total**
0-6 days	2,992.9 (1,794.1–4,581.6)	2,674.3 (1,606.2–3,999.6)	2,838.1 (1,757.7–4,181.0)	1,082.8 (649.1–1,657.6)	913.7 (548.8–1,366.5)	1,996.5 (1,236.5–2,941.2)
7-27 days	2,906.9 (1,739.0–4,446.8)	2,671.5 (1,636.9–4,004.0)	2,792.3 (1,710.7–4,137.9)	3,103.7 (1,856.7–4,747.8)	2,708.6 (1,659.6–4,059.5)	5,812.3 (3,561.0–8,613.3)
28-364 days	1,714.5 (1,050.1–2,640.9)	1,951.9 (1,200.5–2,956.0)	1,830.3 (1,134.4–2,741.8)	28,583.3 (17,506.8–44,029.3)	30,998.8 (19,065.6–46,946.9)	59,582.1 (36,929.0–89,254.7)
1-4 years	691.3 (415.4–1,037.6)	880.7 (525.9–1,334.7)	783.8 (474.7–1,160.6)	46,293.7 (27,818.4–69,483.6)	56,326.9 (33,634.4–85,362.5)	102,620.6 (62,150.7–151,953.8)
5-9 years	845.6 (486.7–1,332.9)	1,207.7 (686.0–2,036.9)	1,022.2 (609.8–1,618.4)	64,178.0 (36,937.7–101,161.8)	87,314.9 (49,599.6–147,267.40	151,492.9 (90,365.6–239,843.6)
10-14 years	386.5 (152.6–830.7)	682.8 (292.0–1,408.5)	530.9 (268.0–955.4)	27,222.1 (10,749.1–58,511.2)	45,685.0 (19,534.9–94,234.1)	72,907.0 (36,804.6–131,213.6)
15-19 years	5.5 (2.8–9.4)	7.3 (3.9–11.9)	6.4 (3.6–10.4)	335.8 (172.2–570.9)	429.7 (231.5–699.3)	765.5 (428.7–1,239.7)
20-24 years	5.4 (2.8–9.1)	7.3 (3.8–12.1)	6.3 (3.5–10.3)	271.4 (140.1–457.8)	367.2 (189.5–609.9)	638.6 (349.9–1,036.0)
25-29 years	5.4 (2.6–9.2)	7.5 (4.2–12.3)	6.5 (3.5–10.5)	209.7 (99.1–356.9)	306.7 (171.3–502.3)	516.4 (281.7–832.8)
30-34 years	5.2 (2.7–9.0)	7.0 (3.8–11.0)	6.1 (3.5–9.7)	160.5 (81.8–278.6)	235.8 (128.6–369.8)	396.3 (227.3–627.5)
35-39 years	4.9 (2.5–8.0)	6.6 (3.8–10.5)	5.8 (3.4–8.9)	129.9 (65.9–213.4)	188.9 (109.5–299.4)	318.7 (187.0–488.9)
40-44 years	4.7 (2.5–7.7)	6.5 (3.5–10.2)	5.6 (3.1–8.6)	101.1 (53.7–166.0)	139.2 (76.0–219.9)	240.3 (133.9–371.7)

45-49 years	4.7 (2.6–7.7)	6.5 (3.6–10.6)	5.6 (3.2–8.8)	75.6 (41.7–124.8)	99.0 (54.9–160.5)	174.6 (99.0–275.3)
50-54 years	4.6 (2.5–7.4)	6.2 (3.2–10.1)	5.4 (3.0–8.3)	55.5 (29.8–88.9)	71.3 (37.3–116.7)	126.7 (71.7–194.7)
55-59 years	4.3 (2.3–6.9)	5.7 (3.2–9.0)	5.0 (2.9–7.7)	38.9 (21.0–63.0)	50.5 (28.8–80.3)	89.4 (53.0–138.3)
60-64 years	4.0 (2.1–6.5)	5.4 (3.1–9.0)	4.7 (2.8–7.5)	28.7 (14.8–46.6)	38.3 (21.9–63.4)	67.0 (40.0–107.3)
65-69 years	3.6 (1.8–6.1)	5.0 (2.7–8.2)	4.3 (2.4–6.9)	20.3 (9.8–34.1)	25.5 (14.0–41.9)	45.7 (26.0–73.5)
70-74 years	3.3 (1.6–5.7)	4.5 (2.3–7.6)	3.9 (2.1–6.2)	13.5 (6.7–23.5)	16.7 (8.5–28.0)	30.2 (16.6–48.7)
75-79 years	2.9 (1.3–4.9)	3.9 (2.0–6.9)	3.4 (1.9–5.6)	7.7 (3.6–13.1)	9.8 (4.9–17.1)	17.5 (9.8–29.0)
80+	2.4 (1.4–4.0)	3.3 (1.8–5.3)	2.8 (1.7–4.5)	5.3 (3.0–8.7)	6.4 (3.4–10.3)	11.7 (7.0–18.4)
Total	331.2 (213.4–495.2)	443.1 (276.2–676.5)	386.7 (248.9–572.6)	171,917.4 (110,736.7–257,023.4)	225,932.7 (140,824.6–344,915.8)	397,850.1 (256,085.2–589,157.8)

Iodine Deficiency						
	**DALY rate per 100,000 (95%UI)**			**Total number of DALYs (95%UI)**		
**Age**	**Male**	**Female**	**Total**	**Male**	**Female**	**Total**
0-6 days	30.6 (14.3–56.8)	55.0 (25.9–101.8)	42.4 (20.1–79.2)	11.1 (5.2–20.5)	18.8 (8.9–34.8)	29.8 (14.2–55.7)
7-27 days	32.6 (15.9–59.5)	58.1 (28.6–106.5)	45.0 (22.3–82.5)	34.8 (16.9–63.5)	58.9 (29.0–108.0)	93.6 (46.5–171.7)
28-364 days	39.4 (19.8–71.4)	65.9 (33.6–117.9)	52.4 (26.8–93.6)	657.3 (330.7–1,190.7)	1,047.3 (533.7–1,872.9)	1,704.6 (873.1–3,046.2)
1-4 years	44.9 (22.2–80.5)	71.9 (38.2–124.6)	58.1 (30.5–100.2)	3,008.2 (1,486.0–5,393.8)	4,600.2 (2,444.4–7,966.7)	7,608.4 (3,989.5–13,121.5)
5-9 years	55.9 (29.4–95.6)	86.9 (45.2–150.2)	71.0 (37.9–122.6)	4,243.0 (2,230.6–7,257.7)	6,283.5 (3,265.7–10,861.2)	10,526.5 (5,622.7–18,162.4)
10-14 years	70.4 (37.8–121.8)	109.7 (58.5–190.4)	89.5 (48.3–155.7)	4,955.2 (2,664.3–8,579.1)	7,336.9 (3,916.6–12,741.9)	12,292.1 (6,632.8–21,378.2)
15-19 years	78.4 (41.7–135.7)	122.7 (64.7–217.8)	100.2 (52.8–175.3)	4,756.9 (2,528.7–8,233.1)	7,200.6 (3,793.4–12,777.0)	11,957.6 (6,304.7–20,920.8)
20-24 years	79.7 (41.9–137.9)	123.6 (64.4–219.1)	101.7 (54.4–178.4)	4,001.7 (2,105.0–6,924.8)	6,233.8 (3,248.9–11,050.9)	10,235.4 (5,473.9–17,962.9)
25-29 years	79.0 (42.5–134.6)	122.4 (64.2–214.7)	101.3 (54.5–173.9)	3,047.5 (1,639.1–5,196.0)	5,000.7 (2,622.6–8,768.7)	8,048.2 (4,331.2–13,810.4)

30-34 years	78.3 (41.5–135.8)	121.2 (62.5–213.3)	100.7 (52.1–176.7)	2,416.3 (1,280.1–4,188.4)	4,071.6 (2,100.7–7,168.2)	6,487.9 (3,356.3–11,387.9)
35-39 years	77.4 (40.8–134.0)	119.9 (63.3–212.6)	99.4 (51.7–174.7)	2,053.0 (1,082.8–3,556.6)	3,413.0 (1,802.6–6,050.1)	5,466.0 (2,841.9–9,606.5)
40-44 years	76.5 (40.4–132.8)	118.5 (60.7–211.0)	97.4 (50.5–172.3)	1,656.9 (875.1–2,877.7)	2,551.7 (1,306.5–4,541.5)	4,208.6 (2,181.6–7,439.9)
45-49 years	76.0 (40.3–131.2)	117.7 (60.0–208.3)	96.2 (50.5–168.4)	1,230.9 (652.4–2,124.7)	1,788.9 (912.2–3,166.1)	3,019.8 (1,585.7–5,288.1)
50-54 years	76.7 (40.9–133.2)	117.6 (60.2–209.4)	96.7 (50.3–170.6)	924.2 (493.2–1,604.7)	1,353.4 (692.2–2,409.2)	2,277.6 (1,185.5–4,018.4)
55-59 years	77.6 (41.3–133.4)	117.1 (59.3–206.9)	97.2 (51.0–169.2)	704.9 (375.1–1,211.8)	1,042.1 (527.5–1,840.5)	1,747.0 (916.3–3,042.6)
60-64 years	77.1 (41.0–133.7)	115.5 (59.3–205.7)	96.1 (51.0–168.5)	555.7 (295.6–963.5)	815.8 (419.0–1,453.6)	1,371.5 (727.9–2,405.3)
65-69 years	77.0 (41.5–131.7)	114.4 (59.5–202.9)	94.9 (50.3–165.8)	429.1 (231.2–733.5)	583.4 (303.6–1,034.3)	1,012.5 (537.0–1,769.2)
70-74 years	75.8 (40.2–127.8)	114.3 (58.4–199.4)	94.0 (50.0–160.8)	311.5 (165.3–525.2)	423.7 (216.5–739.6)	735.2 (390.7–1,257.6)
75-79 years	73.1 (39.0–122.6)	112.0 (57.5–192.5)	91.8 (48.2–155.8)	195.6 (104.4–328.0)	278.5 (142.9–478.8)	474.1 (248.8–804.1)
80+	68.6 (36.2–118.1)	107.4 (55.2–187.8)	87.0 (45.1–151.1)	148.5 (78.2–255.5)	209.2 (107.5–365.6)	357.7 (185.6-621.1)
Total	68.1 (36.8–116.2)	106.5 (56.8–186.7)	87.1 (47.0–151.0)	35,342.1 (19,071.5–60,285.3)	54,312.0 (28,959.4–95,198.5)	89,654.1 (48,328.6–155,368.8)

Other nutritional deficiencies*

	**DALY rate per 100,000 (95%UI)**			**Total number of DALYs (95%UI)**		
**Age**	**Male**	**Female**	**Total**	**Male**	**Female**	**Total**
28-364 days	62.0 (38.1–106.0)	102.3 (50.8–180.8)	81.6 (51.3–124.3)	1,033.9 (634.8–1,767.3)	1,624.0 (806.9–2,871.7)	2,657.9 (1,670.0–4,047.8)
1-4 years	32.3 (19.4–48.1)	49.4 (25.9–85.0)	40.6 (24.8–61.5)	2,163.1 (1,296.7–3,219.9)	3,157.8 (1,658.9–5,433.2)	5,320.8 (3,244.7–8,047.8)
5-9 years	4.7 (2.8–9.1)	7.2 (3.2–13.2)	5.9 (3.6–9.2)	358.7 (215.3–687.9)	522.9 (231.1–956.0)	881.7 (534.8–1,364.8)
10-14 years	3.1 (1.8–6.0)	4.4 (2.1–8.2)	3.7 (2.3–5.8)	216.8 (124.1–420.9)	296.6 (142.3–551.9)	513.4 (321.9–792.1)
15-19 years	5.5 (3.1–11.6)	8.1 (4.7–13.1)	6.8 (4.5–9.8)	332.6 (189.2–706.6)	475.8 (278.2–769.0)	808.4 (538.6–1,163.6)
20-24 years	9.8	10.5	10.1	490.1	527.9	1,017.9

	(5.7–20.3)	(6.1–15.5)	(6.7–14.5)	(284.5–1,020.2)	(307.9–784.0)	(669.8–1,459.9)
25-29 years	10.5 (6.4–19.2)	12.4 (7.2–19.2)	11.5 (7.7–15.8)	406.9 (248.6–739.5)	505.9 (294.7–786.1)	912.8 (608.0–1,251.7)
30-34 years	9.9 (6.4–18.8)	13.2 (7.9–20.1)	11.6 (7.8–15.6)	306.0 (196.4–580.7)	444.2 (265.9–676.5)	750.2 (504.9–1,006.6)
35-39 years	11.6 (7.3–21.0)	17.0 (10.0–25.8)	14.4 (9.6–19.7)	306.6 (192.5–556.0)	484.9 (284.7–733.0)	791.5 (530.1–1,084.6)
40-44 years	16.5 (10.5–32.4)	22.3 (12.8–33.8)	19.4 (12.6–26.2)	357.2 (228.3–702.5)	480.3 (276.4–727.6)	837.5 (545.4–1,133.5)
45-49 years	16.8 (10.4–36.4)	27.8 (15.4–42.4)	22.1 (14.0–31.1)	272.6 (168.0–590.1)	422.7 (233.7–644.6)	695.4 (438.9–977.2)
50-54 years	30.2 (18.2–64.2)	37.4 (21.3–56.1)	33.7 (21.8–47.5)	363.9 (219.6–774.0)	429.8 (244.9–646.0)	793.7 (513.5–1,118.6)
55-59 years	33.2 (19.3–84.6)	50.7 (28.3–75.4)	41.9 (26.3–60.4)	302.0 (175.8–769.1)	451.1 (251.4–671.2)	753.1 (473.6–1,086.1)
60-64 years	31.3 (18.2–89.7)	48.0 (26.5–72.8)	39.6 (25.1–60.1)	225.3 (131.3–646.4)	339.4 (187.6–514.5)	564.8 (358.6–858.1)
65-69 years	42.1 (25.4–124.0)	47.9 (27.8–77.1)	44.9 (29.7–81.0)	234.6 (141.3–691.0)	244.3 (141.8–393.0)	478.9 (317.1–863.7)
70-74 years	51.9 (28.8–163.3)	52.5 (30.0–77.9)	52.2 (34.1–102.8)	213.4 (118.4–671.3)	194.8 (111.2–288.7)	408.2 (266.8–803.9)
75-79 years	57.7 (31.4–185.5)	91.0 (51.8–143.7)	73.7 (47.1–126.3)	154.4 (84.0–496.3)	226.3 (128.9–357.2)	380.6 (243.3–651.7)
80+	72.7 (43.0–235.4)	125.8 (78.1–178.4)	97.8 (64.8–168.4)	157.2 (93.0–509.2)	244.9 (152.0–347.3)	402.1 (266.5–692.2)
Total	15.2 (10.6–28.5)	21.7 (13.1–31.4)	18.4 (12.9–23.9)	7,895.6 (5,501.9–14,770.5)	11,073.6 (6,687.8–16,005.0)	18,969.1 (13,303.1–24,615.7)

* Thiamine, niacin, other B group vitamins, ascorbic acid, vitamin D, other vitamin, dietary calcium, dietary selenium, dietary zinc, and other nutrient element deficiencies.

Table 2: Age and sex specific prevalence of dietary iron, vitamin A, and iodine deficiencies in Ethiopia, 2017

Dietary iron deficiency						
	Prevalence rate per 100,000 (95%UI)			Total number of prevalent cases (95%UI)		
Age	Male	Female	Total	Male	Female	Total
0–6 days	7,370.8 (6,633.3–8,087.2)	6,772.1 (6,229.9–7,314.6)	7,080.0 (6,588.0–7,552.5)	2,666.8 (2,400.0–2,926.0)	2,313.7 (2,128.5–2,499.1)	4,980.5 (4,634.4–5,312.9)
7–27 days	9,180.0 (8,268.4– 10,111.3)	6,750.8 (6,184.4–7,324.7)	7,996.8 (7,430.5–8,551.6)	9,801.3 (8828.0–10795.8)	6,844.5 (6,270.2–7,426.4)	16,645.8 (15,467.1–17,800.8)
28–364 days	31,222.5 (28,282.6–34,278.2)	20,557.7 (18,891.6–22,172.3)	26,019.5 (24,129.9–27,795.8)	520,536.1 (471,522.6–571,479.9)	326,491.8 (300,031.5–352,133.9)	847,027.9 (785,514.2–904,850.3)
1–4 years	28,467.6 (26,738.4–30,396.2)	24,365.2 (23,029.4–25,692.4)	26,463.6 (25,297.8–27,577.6)	1,906,392.5 (1,790,594.7–2,035,544.3)	1,558,305.0 (1,472,871.7–1,643,186.2)	3,464,697.5 (3,312,068.0–3,610,551.1)
5–9 years	18,039.9 (14,159.2–22,479.8)	12,882.1 (9,407.3–17,091.0)	15,523.5 (12,673.2–18,819.3)	1,369,145.0 (1,074,615.5–1,706,115.4)	931,376.4 (680,148.0–1,235,681.7)	2,300,521.4 (1,878,114.6–2,788,935.6)
10–14 years	7,629.5 (3,630.2–13,933.6)	6,091.9 (2,990.0–12,575.5)	6,880.5 (4,069.1–10,884.6)	537,381.1 (255,691.8–981,407.5)	407,577.1 (200,044.9–841,360.1)	944,958.2 (558,849.2–1,494,878.4)
15–19 years	14,715.6 (11,710.2–18,285.6)	16,029.8 (14,191.1–18,024.5)	15,361.6 (13,599.7–17,283.3)	892,883.9 (710,528.1–1,109,499.0)	940,339.3 (832,472.4–1,057,347.4)	1,833,223.2 (1,622,962.0–2,062,548.6)
20–24 years	8,265.9 (6,339.4–10,333.6)	18,303.6 (16,090.9–20,750.3)	13,295.4 (11,714.2–14,821.3)	415,142.8 (318,388.6–518,992.0)	923,198.1 (811,593.3–1,046,608.5)	1,338,341.0 (1,179,175.1–1,491,933.8)
25–29 years	8,953.2 (6,647.6–11,671.0)	14,765.2 (13,141.4–16,293.0)	11,941.4 (10,680.3–13,494.7)	345,529.3 (256,550.6–450,416.1)	603,007.2 (536,691.7–665,404.4)	948,536.5 (848,366.6–1,071,922.5)
30–34 years	6,899.4 (5,163.9–9,252.6)	16,257.4 (14,468.2–18,201.0)	11,778.6 (10,475.2–13,271.0)	212,836.3 (159,298.8–285,427.8)	546,346.4 (486,220.7–611,664.2)	759,182.8 (675,171.8–855,375.1)
35–39 years	10,949.9 (8,509.4–13,686.5)	13,496.3 (11,955.6–15,166.8)	12,267.8 (10,928.0–13,837.7)	290,557.0 (225,797.7–363,170.3)	384,144.9 (340,291.9–431,691.3)	674,701.9 (601,017.5–761,043.2)
40–44 years	11,575.3 (9,208.2–14,142.7)	11,774.4 (10,225.5–13,454.6)	11,674.5 (10,262.6–13,155.3)	250,809.6 (199,519.0–306,437.9)	253,440.7 (220,100.1–289,605.5)	504,250.2 (443,262.7–568,205.6)
45–49 years	13,960.9 (10,766.3–17,283.4)	12,582.3 (10,642.2–14,629.9)	13,293.3 (11,464.2–15,435.3)	226,054.7 (174,327.7–279,853.0)	191,273.8 (161,781.3–222,400.7)	417,328.5 (359,905.3–484,574.0)
50–54 years	14,438.0 (11,783.9–17,828.4)	8,186.1 (3,709.3–14,797.9)	11,384.2 (8,657.6–14,797.7)	173,974.4 (141,993.2–214,827.6)	94,188.9 (42,678.7–170,264.7)	268,163.3 (203,936.0–348,571.5)

55–59 years	12,737.1 (9,787.1–16,268.0)	8,969.6 (4,669.0–15,430.2)	10,873.3 (8,150.2–14,369.5)	115,728.1 (88,924.1–147,809.2)	79,790.6 (41,534.4–137,262.4)	195,518.6 (146,553.0–258,386.5)
60–64 years	10,193.3 (4,639.3–18,799.9)	7,325.2 (3,609.2–13,381.1)	8,773.4 (5,113.4–14,526.0)	73,463.8 (33,435.6–135,492.0)	51,759.4 (25,502.3–94,550.4)	125,223.2 (72,982.8–207,329.7)
65–69 years	5,992.3 (2,571.0–11,270.3)	9,936.3 (5,104.4–16,292.2)	7,876.9 (4,885.4–12,068.9)	33,384.6 (14,323.8–62,789.2)	50,657.1 (26,023.5–83,060.8)	84,041.7 (52,124.7–128,768.4)
70–74 years	9,717.9 (4,983.0–17,233.1)	8,114.6 (4,269.7–13,646.4)	8,957.6 (5,522.1–13,353.8)	39,954.0 (20,487.0–70,851.8)	30,092.2 (15,833.7–50,606.1)	70,046.2 (43,181.6–104,423.8)
75–79 years	10,233.9 (4,856.6–18,611.8)	3,834.5 (1,377.9–9,028.1)	7,151.2 (3,714.9–11,968.2)	27,378.3 (12,992.8–49,791.6)	9,534.4 (3,426.2–2,2448.1)	36,912.7 (19,175.6–61,776.6)
80+	6,918.7 (4,407.4–10,106.7)	6,023.4 (2,880.9–11,864.1)	6494.6 (4,479.1–9,379.9)	14,969.3 (9,536.2–21,866.9)	11,727.4 (5,609.1–23,099.2)	26,696.7 (18,411.4–38,556.7)
Total	14,371.1 (13,233.9–15,742.2)	14,519.1 (13,681.5-15,527.5)	14,444.4 (13,714.3–15,383.7)	7,458,588.9 (6,868,361.1–8,170,194.6)	7,402,409.0 (6,975,372.8–7,916,556.4)	14,860,997.8 (14,109,816.8–15,827,351.6)

Vitamin A Deficiency

	Prevalence rate per 100,000 (95%UI)			Total number of prevalent cases (95%UI)		
**Age**	**Male**	**Female**	**Total**	**Male**	**Female**	**Total**
0-6 days	84,861.9 (62,874.7–96,938.1)	84,213.8 (62,249.1–96,401.4)	84,547.1 (62,982.6–96,665.2)	30,703.6 (22,748.5–35,072.8)	28,772.2 (21,267.8–32,936.1)	59,475.7 (44,305.9–68,000.4)
7-27 days	81,624.6 (60,543.3–93,695.9)	80,691.2 (59,851.0–92,413.4)	81,169.9 (60,275.8–92,788.2)	87,149.6 (64,641.4–100,038.0)	81,811.0 (60,681.6–93,695.8)	168,960.6 (125,468.1–193,144.8)
28-364 days	53,993.8 (41,819.9–63,811.4)	53,777.5 (41,059.5–63,270.2)	53,888.3 (41,476.0–63,440.6)	900,174.3 (697,213.9–1,063,851.7)	854,080.1 (652,096.4–1,004,841.0)	1,754,254.4 (1,350,191.0–2,065,216.6)
1-4 years	31,700.7 (25,165.0–38,024.0)	31,324.8 (25,370.9–37,676.7)	31,517.1 (25,392.5–37,881.8)	2,122,903.5 (1,685,229.3–2,546,358.0)	2,003,412.8 (1,622,626.2–2,409,653.7)	4,126,316.3 (3,324,460.6–4,959,600.2)
5-9 years	34,821.6 (29,818.9–40,797.5)	36,283.9 (30,260.3–43,807.9)	35,535.0 (30,315.3–41,731.7)	2,642,794.8 (2,263,115.7–3,096,337.1)	2,623,337.3 (2,187,825.3–3,167,325.3)	5,266,132.1 (4,492,601.2–6,184,456.8)
10-14 years	26,028.1 (22,315.8–31,050.9)	27,349.8 (23,219.6–34,922.2)	26,672.0 (22,988.4–31,014.2)	1,833,284.7 (1,571,810.4–2,187,062.5)	1,829,827.9 (1,553,496.6–2,336,458.5)	3,663,112.6 (3,157,219.1–4,259,473.0)
15-19 years	24,761.2 (21,717.3–27,908.5)	25,124.6 (22,124.3–28,352.2)	24,939.8 (21,896.7–27,913.9)	1,502,413.0 (1,317,723.0–1,693,380.9)	1,473,855.8 (1,297,848.3–1,663,190.2)	2,976,268.9 (2,613,104.5–3,331,187.4)
20-24 years	24,360.1 (21,505.7–27,279.9)	24,744.8 (21,916.1–27,688.5)	24,552.8 (21,668.2–27,287.1)	1,223,451.7 (1,080,093.8–1,370,096.5)	1,248,080.1 (1,105,408.6–1,396,555.7)	2,471,531.8 (2,181,162.6–2,746,762.5)
25-29 years	23,936.7 (21,157.4–26,624.5)	24,385.5 (21,650.3–27,112.8)	24,167.4 (21,414.0–26,711.5)	923,784.9 (816,523.8–1,027,514.0)	995,900.1 (884,194.8–1,107,284.6)	1,919,685.0 (1,700,970.4–2,121,767.4)

30-34 years	23,551.1 (20,947.4–26,043.1)	24,106.7 (21,518.4–26,631.6)	23,840.8 (21,257.7–26,226.4)	726,511.5 (646,191.3–803,386.9)	810,131.4 (723,150.7–894,985.1)	1,536,642.8 (1,370,150.1–1,690,404.6)
35-39 years	23,241.3 (20,673.2–25,582.3)	23,876.1 (21,387.1–26,273.6)	23,569.8 (21,081.4–25,845.9)	616,709.0 (548,563.7–678,827.2)	679,582.2 (608,738.1–747,822.5)	1,296,291.2 (1,159,433.5–1,421,471.2)
40-44 years	22,987.0 (20,517.9–25,183.2)	23,641.5 (21,276.4–25,955.9)	23,313.2 (20,943.4–25,469.9)	498,074.0 (444,573.3–545,659.2)	508,875.4 (457,967.0–558,692.1)	1,006,949.3 (904,592.1–1,100,104.4)
45-49 years	22,744.5 (20,381.2–24,900.3)	23,391.3 (21,036.3–25,646.8)	23,057.7 (20,735.9–25,164.8)	368,279.7 (330,012.8–403,186.4)	355,590.7 (319,790.5–389,878.7)	723,870.4 (650,981.0–790,019.7)
50-54 years	22,488.5 (20,225.8–24,597.3)	23,100.6 (20,767.9–25,327.4)	22,787.5 (20,538.7–24,920.7)	270,980.2 (243,715.6–296,391.1)	265,795.5 (238,955.8–291,416.4)	536,775.8 (483,803.3–587,025.0)
55-59 years	22,204.1 (19,960.6–24,348.3)	22,786.3 (20,441.3–24,979.9)	22,492.1 (20,259.7–24,645.6)	201,744.5 (181,360.4–221,225.9)	202,699.6 (181,839.2–222,213.1)	404,444.1 (364,302.1–443,167.2)
60-64 years	21,908.0 (19,648.9–24,089.3)	22,503.9 (20,107.0–24,755.7)	22,203.0 (19,959.0–24,382.0)	157,892.0 (141,610.9–173,612.7)	159,011.2 (142,075.2–174,922.2)	316,903.2 (284,875.3–348,004.7)
65-69 years	21,617.5 (19,287.0–23,792.8)	22,248.3 (19,795.524,549.5)	21,918.9 (19,527.3–24,153.1)	120,435.9 (107,451.9–132,554.7)	113,426.3 (100,921.4–125,158.4)	233,862.3 (208,344.4–257,699.1)
70-74 years	21,370.8 (19,022.3–23,644.9)	22,037.9 (19,518.6–24,423.9)	21,687.2 (19,279.1–23,992.9)	87,863.6 (78,207.9–97,213.5)	81,725.1 (72,382.3–90,573.1)	169,588.7 (150,757.9–187,619.3)
75-79 years	21,118.8 (18,635.0–23,433.5)	21,806.8 (19,175.8–24,264.0)	21,450.2 (18,917.0–23,796.1)	56,498.5 (49,853.6–62,690.9)	54,221.9 (47,680.0–60,331.5)	110,720.4 (97,644.5–122,829.0)
80+	20,847.7 (18,210.9–23,258.7)	21,501.8 (18,721.7–24,031.0)	21,157.5 (18,486.0–23,560.8)	45,105.8 (39,401.0–50,322.4)	41,863.6 (36,450.7–46,787.7)	86,969.4 (75,988.1–96,848.2)
Total	27,778.0 (25,001.4–30,680.5)	28,267.6 (25,501.1–31,227.7)	28,020.7 (25,351.0–30,861.5)	14,416,754.8 (12,975,705.5–15,923,120.3)	14,412,000.0 (13,001,484.1–15,921,175.2)	28,828,754.9 (26,082,128.8–31,751,529.9)

Iodine Deficiency

	**Prevalence rate per 100,000 (95%UI)**			**Total number of prevalent cases (95%UI)**		
**Age**	**Male**	**Female**	**Total**	**Male**	**Female**	**Total**
0-6 days	2,760.8 (2,145.1–3,512.9)	4,963.8 (3,860.4–6,215.1)	3,830.8 (2,986.9–4,808.4)	998.9 (776.1–1,271.0)	1,695.9 (1,318.9–2,123.4)	2,694.8 (2,101.2–3,382.5)
7-27 days	2,766.4 (2,148.1–3,514.6)	4,972.8 (3,866.2–6,222.4)	3,841.1 (2,997.4–4,820.2)	2,953.7 (2,293.5–3,752.5)	5,041.8 (3,919.9–6,308.8)	7,995.5 (6,239.4–10,033.7)
28-364 days	2,793.7 (2,179.1–3,526.3)	5,007.8 (3,908.8–6,269.1)	3,873.9 (3,032.2–4,856.1)	46,576.3 (36,329.5–58,790.5)	79,531.9 (62,078.8–99,564.0)	126,108.2 (98,709.9–158,084.5)
1-4 years	2,958.3 (2,374.2–3,677.3)	5,276.1 (4,214.2–6,477.6)	4,090.5 (3,282.9–5,050.0)	198,108.5 (158,993.9–246,257.6)	337,436.9 (269,526.1–414,282.2)	535,545.4 (429,812.1–661,158.7)
5-9 years	3,699.7 (3,152.0–4,376.5)	6,514.4 (5,581.7–7,652.6)	5,072.9 (4,353.9–5,945.3)	280,788.8 (239,220.1–332,156.6)	470,989.9 (403,561.3–553,286.6)	751,778.7 (645,235.2–881,060.8)

10-14 years	4,957.9 (4,392.8–5,605.3)	8,611.4 (7,679.4–9,707.0)	6,737.7 (6,006.1–7,599.9)	349,211.0 (309,409.1–394,805.3)	576,141.5 (513,787.7–649,440.4)	925,352.5 (824,872.2–1,043,772.2)
15-19 years	5,724.4 (5,108.5–6,481.7)	9,866.1 (8,824.8–11,110.8)	7,760.3 (6,953.5–8,737.6)	347,331.5 (309,963.6–393,284.9)	578,760.7 (517,676.9–651,781.8)	926,092.3 (829,816.5–1,042,729.0)
20-24 years	5,885.0 (5,248.1–6,665.7)	10,105.6 (9,057.3–11,377.3)	7,999.8 (7,180.5–8,990.6)	295,567.8 (263,576.5–334,777.3)	509,707.0 (456,832.8–573,851.0)	805,274.8 (722,806.6–905,014.1)
25-29 years	5,920.3 (5,286.8–6,705.7)	10,137.2 (9,090.0–11,394.9)	8,088.4 (7,260.8–9,081.4)	228,481.5 (204,031.7–258,793.1)	414,000.2 (371,235.2–465,365.2)	642,481.7 (576,746.9–721,358.6)
30-34 years	5,953.4 (5,314.7–6,730.3)	10,166.4 (9,119.2–11,413.6)	8,150.1 (7,316.4–9,143.5)	183,653.7 (163,949.3–207,620.0)	341,653.6 (306,459.6–383,567.8)	525,307.3 (471,573.3–589,336.2)
35-39 years	5,984.8 (5,350.4–6,757.1)	10,194.2 (9,145.9–11,434.8)	8,163.2 (7,331.0–9,155.2)	158,805.9 (141,973.8–179,300.9)	290,155.3 (260,318.1–325,468.9)	448,961.3 (403,192.5–503,514.5)
40-44 years	6,014.4 (5,382.1–6,784.3)	10,220.4 (9,171.6–11,463.1)	8,110.4 (7,282.8–9,091.5)	130,317.9 (116,616.9–147,000.0)	219,990.3 (197,415.7–246,739.9)	350,308.2 (314,562.0–392,681.8)
45-49 years	6,043.3 (5,408.2–6,814.20	10,245.7 (9,196.6–11,484.2)	8,078.2 (7,251.7–9,051.0)	97,852.4 (87,569.3–110,335.8)	155,753.9 (139,805.1–174,580.2)	253,606.3 (227,659.4–284,145.6)
50-54 years	6,074.6 (5,442.4–6,847.0)	10,272.4 (9,222.2–11,499.4)	8,125.0 (7,295.9–9,099.2)	73,197.3 (65,579.0–82,503.9)	118,193.9 (106,110.3–132,311.8)	191,391.3 (171,860.3–214,338.2)
55-59 years	6,104.9 (5,471.5–6,872.5)	10,295.9 (9,246.8–11,520.4)	8,178.2 (7,350.7–9,159.5)	55,468.7 (49,713.7–62,442.5)	91,589.1 (82,256.9–102,482.1)	147,057.8 (132,177.0–164,702.3)
60-64 years	6,131.2 (5,499.9–6,896.3)	10,316.2 (9,266.1–11,539.8)	8,203.0 (7,376.0–9,183.8)	44,187.8 (39,638.1–49,702.0)	72,893.5 (65,473.9–81,539.2)	117,081.3 (105,278.1–131,080.0)
65-69 years	6,157.4 (5,530.2–6,924.4)	10,338.1 (9,291.8–11,559.0)	8,155.0 (7,335.0–9,127.3)	34,304.0 (30,810.1–38,577.5)	52,705.4 (47,371.2–58,930.2)	87,009.4 (78,260.3–97,383.3)
70-74 years	6,178.3 (5,545.0–6,951.3)	10,362.1 (9,326.9–11,587.8)	8,162.4 (7,345.2–9,133.4)	25,401.4 (22,797.7–28,579.6)	38,426.7 (34,587.7–42,972.1)	63,828.1 (57,437.7–71,420.9)
75-79 years	6,191.9 (5,555.5–6,968.4)	10,378.0 (9,341.9–11,604.4)	8,208.3 (7,389.7–9,176.8)	16,564.9 (14,862.6–18,642.4)	25,804.4 (23,228.3–28,853.9)	42,369.3 (38,143.8–47,368.4)
80+	6,204.7 (5,577.5–6,982.7)	10,391.3 (9,351.2–11,610.8)	8,187.6 (7,378.3–9,158.0)	13,424.3 (12,067.4–15,107.7)	20,231.5 (18,206.5–22,606.0)	33,655.9 (30,329.2–37,644.4)
Total	4,977.3 (4,407.1–5,616.0)	8,631.5 (7,701.2–9,731.0)	6,788.1 (6,051.7–7,633.8)	2,583,196.5 (2,287,277.8–2,914,674.6)	4,400,703.5 (3,926,389.4–4,961,283.8)	6,983,900.0 (6,226,204.6–7,853,909.2)

Table 3: Age and sex specific incidence of vitamin A and iodine deficiencies in Ethiopia, 2017

**Vitamin A deficiency**						
	**Incidence rate per 100,000 (95%UI)**			**Number of new cases (95%UI)**		
**Age**	**Male**	**Female**	**Both**	**Male**	**Female**	**Both**
0-6 days	4,593.1 (1,025.5–10,837.5)	4,939.1 (1,108.6–11,673.7)	4,761.1 (1,109.7–11,236.7)	1,661.8 (371.0–3,921.1)	1,687.5 (378.8–3,988.4)	3,349.3 (780.7–7,904.6)
7-27 days	5,704.2 (2,204.5–11,744.3)	6,132.3 (2,387.7–12,605.8)	5,912.7 (2,365.0–12,093.7)	6,090.3 (2,353.7–12,539.2)	6,217.4 (2,420.9–12,780.8)	12,307.8 (4,922.9–25,173.8)
28-364 days	14,908.8 (11,222.3–19,230.6)	15,796.8 (12,028.1–20,165.9)	15,342.0 (11,722.1–19,525.5)	248,556.1 (187,096.8–320,608.1)	250,881.3 (191,027.7–320,269.1)	499,437.3 (381,596.6–635,625.1)
1-4 years	24,089.5 (21,216.4–26,650.3)	24,513.1 (21,829.7–27,080.4)	24,296.4 (21,603.6–26,817.3)	1,613,203.0 (1,420,798.0–1,784,694.2)	1,567,763.4 (1,396,140.2–1,731,958.7)	3,180,966.5 (2,828,417.3–3,511,011.6)
5-9 years	22,997.8 (20,387.7–25,325.9)	22,820.1 (20,368.1–25,183.2)	22,911.1 (20,367.2–25,233.8)	1,745,426.9 (1,547,330.4–1,922,118.0)	1,649,897.3 (1,472,616.5–1,820,750.6)	3,395,324.2 (3,018,337.4–3,739,538.2)
10-14 years	23,797.7 (21,063.1–26,138.1)	23,510.3 (20,815.0–25,950.1)	23,657.7 (21,032.6–25,970.9)	1,676,184.9 (1,483,577.0–1,841,031.5)	1,572,946.8 (1,392,617.9–1,736,180.4)	3,249,131.7 (2,888,604.8–3,566,826.8)
15-19 years	23,925.8 (21,193.8–26,213.1)	23,623.9 (21,011.4–26,023.4)	23,777.4 (21,203.7–26,044.5)	1,451,722.7 (1,285,957.3–1,590,511.8)	1,385,821.0 (1,232,567.1–1,526,575.9)	2,837,543.7 (2,530,401.7–3,108,096.4)
20-24 years	23,861.1 (21,220.1–26,143.3)	23,552.5 (20,984.2–25,937.8)	23,706.5 (21,123.6–25,983.7)	1,198,389.9 (1,065,747.0–1,313,011.5)	1,187,943.2 (1,058,403.8–1,308,253.7)	2,386,333.1 (2,126,341.8–2,615,563.3)
25-29 years	23,772.8 (21,188.4–26,045.2)	23,481.8 (20,974.3–25,838.9)	23,623.2 (21,030.5–25,861.4)	917,459.3 (817,719.2–1,005,157.4)	958,991.6 (856,587.0–1,055,257.7)	1,876,450.9 (1,670,506.5–2,054,242.7)
30-34 years	23,694.8 (21,133.9–25,992.7)	23,451.0 (20,956.6–25,808.9)	23,567.7 (20,984.8–25,787.5)	730,945.7 (651,946.7–801,830.7)	788,095.8 (704,269.2–867,337.4)	1,519,041.5 (1,352,564.1–1,662,117.4)
35-39 years	23,656.0 (20,963.5–25,987.6)	23,446.1 (20,947.3–25,791.0)	23,547.4 (20,898.6–25,776.6)	627,711.4 (556,266.6–689,582.2)	667,345.7 (596,221.8–734,086.8)	1,295,057.1 (1,149,379.0–1,417,660.6)
40-44 years	23,648.6 (20,996.3–26,005.2)	23,437.7 (20,860.8–25,754.0)	23,543.5 (20,866.9–25,747.4)	512,408.6 (454,939.4–563,470.9)	504,489.1 (449,021.9–554,345.3)	1,016,897.6 (901,287.5–1,112,090.3)

45-49 years	23,643.7 (20,930.8–26,050.2)	23,419.2 (20,776.8–25,729.2)	23,535.0 (20,859.7–25,800.7)	382,839.3 (338,911.2–421,805.2)	356,014.5 (315,846.2–391,131.4)	738,853.8 (654,866.4–809,984.4)
50-54 years	23,625.9 (20,897.2–26,058.3)	23,371.7 (20,724.5–25,735.0)	23,501.7 (20,809.2–25,822.7)	284,685.8 (251,806.2–313,995.9)	268,914.6 (238,455.6–296,106.8)	553,600.4 (490,176.5–608,272.9)
55-59 years	23,587.0 (20,827.9–26,052.7)	23,303.4 (20,575.3–25,722.0)	23,446.7 (20,715.4–25,811.3)	214,309.4 (189,240.2–236,712.6)	207,299.1 (183,031.5–228,814.6)	421,608.6 (372,495.7–464,127.2)
60-64 years	23,534.4 (20,645.8–26,054.3)	23,247.0 (20,438.6–25,727.5)	23,392.1 (20,655.1–25,826.6)	169,613.5 (148,795.4–187,774.6)	164,262.1 (144,417.9–181,788.9)	333,875.6 (294,810.3–368,623.0)
65-69 years	23,479.1 (20,512.5–26,099.3)	23,204.9 (20,346.3–25,766.7)	23,348.0 (20,568.5–25,848.3)	130,807.0 (114,279.7–145,404.7)	118,303.0 (103,729.6–131,363.5)	249,110.0 (219,454.3–275,786.1)
70-74 years	23,449.5 (20,460.6–26,118.6)	23,189.8 (20,304.2–25,814.9)	23,326.4 (20,459.3–25,874.2)	96,409.9 (84,121.5–107,383.7)	85,996.7 (75,295.6–95,731.4)	182,406.7 (159,986.9–202,330.0)
75-79 years	23,411.9 (20,305.8–26,134.9)	23,159.1 (20,219.6–25,863.1)	23,290.1 (20,327.9–25,893.5)	62,633.1 (54,323.6–69,917.9)	57,584.3 (50,275.4–64,307.7)	120,217.4 (104,927.1–133,655.3)
80+	23,399.1 (20,224.3–26,185.7)	23,126.1 (20,024.9–25,957.6)	23,269.8 (20,215.4–25,924.9)	50,626.2 (43,757.2–56,655.2)	45,025.9 (38,987.9–50,538.9)	95,652.0 (83,096.9–106,566.2)
Total	23,355.9 (21,069.8–25,208.8)	23,233.7 (21,010.9–25,309.5)	23,295.3 (21,104.5–25,199.4)	12,121,684.9 (10,935,166.0–13,083,321.8)	11,845,480.3 (10,712,222.0–12,903,809.4)	23,967,165.2 (21,713,099.5—25,926,169.5)

Iodine deficiency

	**Incidence rate**			**Total incident cases**		
**Age**	**Male**	**Female**	**Both**	**Male**	**Female**	**Both**
0-6 days	0.3 (0.2–0.4)	0.5 (0.4–0.6)	0.4 (0.3–0.5)	0.1 (0.1–0.1)	0.2 (0.1–0.2)	0.3 (0.2–0.3)
7-27 days	1.5 (1.1–1.8)	2.5 (1.9–3.1)	2 (1.5–2.4)	1.6 (1.2–1.9)	2.5 (2.0–3.1)	4.1 (3.2–5.1)
28-364 days	16.6 (12.8–20.3)	28 (21.8–34.3)	22.2 (17.4–27.3)	276.1 (213.2–337.7)	445.1 (346.7–544.3)	721.2 (565.0–888.5)
1-4 years	92.1 (71.2–112.6)	155.7 (121.4–190.4)	123.2 (96.5–151.7)	6,169.3 (4,767.4–7,543.5)	9,956.7 (7,762.4–12,175.2)	16,126.0 (12,638.2–19,860.0)
5-9 years	228.4 (176.9–278.8)	383.6 (300.1–468.1)	304.1 (238.9–373.5)	17,332.5 (13,428.0–21,159.5)	27,730.9 (21,695.8–33,844.5)	45,063.4 (35,409.5–55,344.7)
10-14 years	227.1 (177.4–275.8)	375.9 (296.2–456.2)	299.6 (236.8–366.1)	15,996.4 (12,494.0–19,427.6)	25,151.5 (19,816.7–30,519.8)	41,147.9 (32,528.7–50,276.7)
15-19 years	80.5 (64.6–96.1)	128.6 (102.9–154.7)	104.1 (83.6–125.5)	4,881.5 (3,922.5–5,831.5)	7,542.7 (6,036.0–9,072.4)	12,424.2 (9,975.4–14,972.5)
20-24 years	8 (5.8–10.5)	7.6 (5.6–10)	7.8 (5.7–10.3)	399.3 (293.2–525.9)	383.1 (282.1–505.6)	782.4 (577.2–1,032.6)

25-29 years	7.6 (5.7–9.9)	7.3 (5.4–9.5)	7.4 (5.5–9.7)	293.3 (218.9–383.5)	296.3 (221.3–388.7)	589.6 (440.8–773.4)
30-34 years	7.2 (5.5–9.4)	6.9 (5.2–9)	7.1 (5.3–9.2)	223.6 (169.0–290.3)	232.4 (175.8–302.3)	456.1 (344.8–591.8)
35-39 years	6.9 (5.3–8.9)	6.6 (5.1–8.5)	6.7 (5.2–8.7)	183.1 (140.4–235.8)	187.2 (143.8–240.9)	370.3 (284.0–476.5)
40-44 years	6.5 (5.1–8.4)	6.2 (4.9–7.9)	6.4 (5–8.1)	141.9 (110.6–181.1)	134.3 (105.8–170.7)	276.2 (215.8–351.9)
45-49 years	6.2 (4.9–7.8)	5.9 (4.7–7.4)	6.1 (4.8–7.6)	100.4 (79.6–126.1)	89.7 (71.1–112.8)	190.1 (151.3–239.8)
50-54 years	5.8 (4.7–7.3)	5.6 (4.5–6.9)	5.7 (4.6–7.1)	70.5 (57.0–87.4)	64.0 (51.7–79.3)	134.5 (108.6–166.4)
55-59 years	5.5 (4.5–6.7)	5.2 (4.3–6.4)	5.4 (4.4–6.5)	50.0 (40.9–61.2)	46.5 (38.0–56.5)	96.5 (79.0–117.7)
60-64 years	5.2 (4.2–6.2)	4.9 (4.1–5.9)	5 (4.2–6.1)	37.1 (30.6–44.8)	34.5 (28.7–41.6)	71.7 (59.3–86.7)
65-69 years	4.8 (4.0–5.8)	4.6 (3.8–5.5)	4.7 (3.9–5.6)	26.8 (22.2–32.1)	23.2 (19.4–28.0)	50.0 (41.7–60.0)
70-74 years	4.5 (3.7–5.4)	4.2 (3.5–5.1)	4.3 (3.6–5.2)	18.3 (15.1–22.1)	15.6 (13.0–18.9)	34.0 (28.1–41.0)
75-79 years	4.1 (3.3–5.1)	3.9 (3.1–4.8)	4 (3.2–4.9)	11.0 (8.8–13.7)	9.6 (7.8–11.9)	20.6 (16.6–25.6)
80+	3.6 (2.8–4.8)	3.4 (2.6–4.5)	3.5 (2.7–4.6)	7.8 (5.9–10.3)	6.6 (5.0–8.7)	14.5 (11.0–18.9)
Total	199.8(177.4–226.1)	334.1 (298.7–376.1)	266.4 (237.7–299.1)	103,718.8 (92,080.8–117,327.2)	170,360.7 (152,267.3–191,770.4)	274,079.5 (244,558.6–307,693.8)

Figure 1: Rank of all causes of years lived with disabilities (YLDs) in Ethiopia, in 1990 and 2017 (Source: https://vizhub.healthdata.org/gbd-compare/)

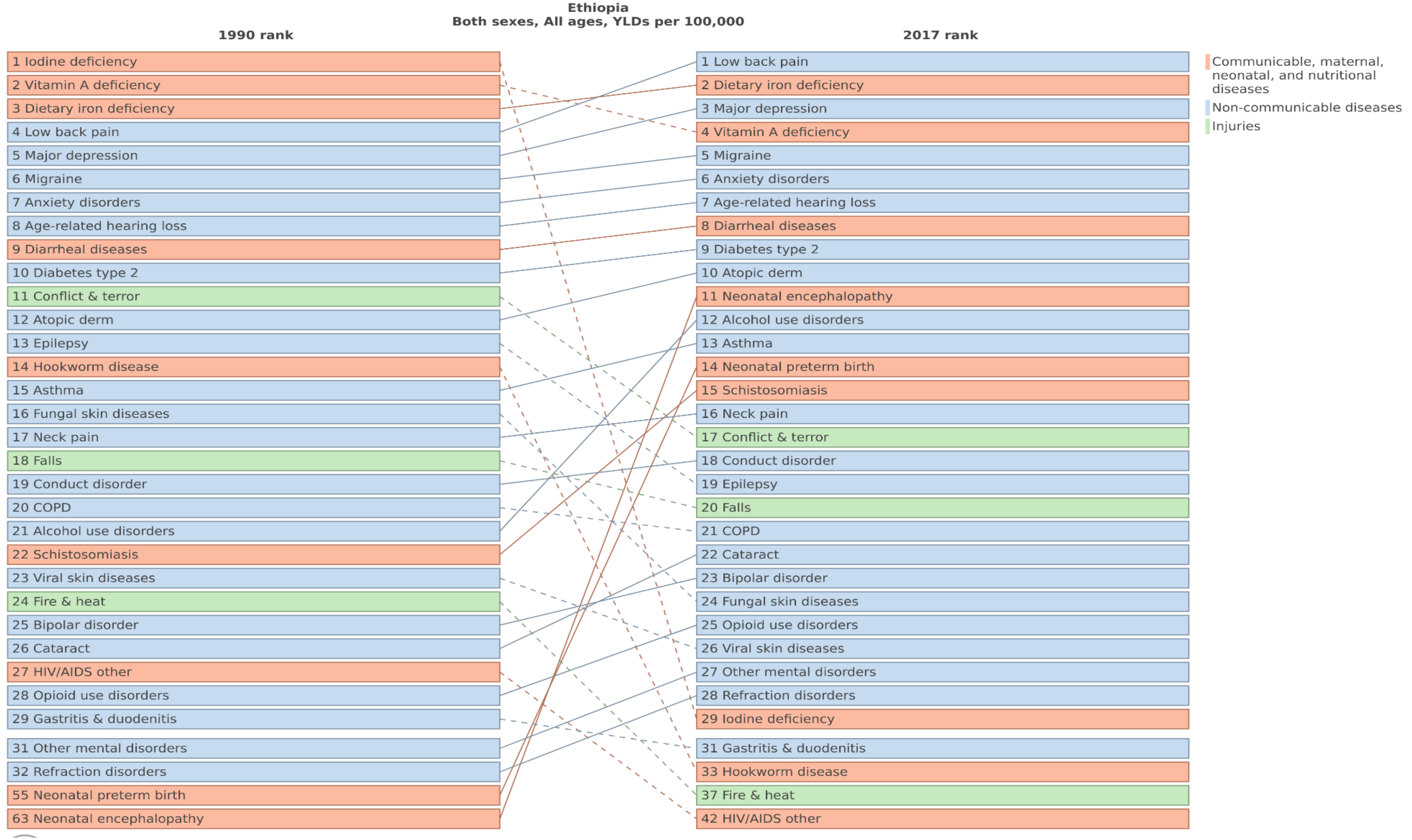

Supplement: Supplemental Material [file ZGHA_A_1776507_SM5421.pdf]
